# Real-world use of inhaled corticosteroid/formoterol as needed in adults with mild asthma: the PRIME study

**DOI:** 10.1183/23120541.00174-2024

**Published:** 2024-09-30

**Authors:** Guy Brusselle, Francesco Blasi, Christian Gessner, Piotr Kuna, Peter Wark, Glauco Cappellini, Emilie Oosterom, Marielle Van Der Deijl, Enrica Bucchioni, Eva Topole

**Affiliations:** 1 Department of Respiratory Medicine, Ghent University Hospital, Ghent, Belgium; 2 Pulmonology and Cystic Fibrosis Unit, Foundation IRCCS Ca' Granda Ospedale Maggiore Policlinico di Milano, Milan, Italy; 3 Department of Pathophysiology and Transplantation, University of Milan, Milan, Italy; 4 University of Leipzig, Institute for Clinical Immunology, Leipzig, Germany; 5 Department of Internal Medicine, Asthma and Allergy, Norbert Barlicki Memorial University Hospital No. 1, Medical University of Łódź, Łódź, Poland; 6 Central Clinical School, Monash University, Melbourne, Australia; 7 AIRMED, Alfred Hospital, Melbourne, Australia; 8 Global Clinical Development, Chiesi Farmaceutici SpA, Parma, Italy; 9 Global Medical Affairs, Chiesi Farmaceutici SpA, Parma, Italy

## Abstract

**Introduction:**

Inhaled corticosteroid/formoterol fumarate (ICS/FF) as needed is recommended by the Global Initiative for Asthma (GINA) as sole therapy in adults with mild asthma, with low-dose maintenance ICS plus short-acting β_2_-agonist (SABA) as an alternative. SABA alone is no longer recommended. Given these changes in recommendations, the observational PRIME study aimed to describe real-world treatment patterns in mild asthma in Europe.

**Methods:**

Adults with asthma receiving low-dose maintenance ICS, or as needed ICS/FF or SABA were followed for 6 months. Data collected included Asthma Control Test (ACT), Asthma Control Questionnaire 5-item (ACQ-5), forced expiratory volume in 1 s (FEV_1_) and asthma exacerbations.

**Results:**

The study was conducted in 883 patients in Germany, Italy, Poland and Spain; 833 (94.3%) completed follow-up. At enrolment, 32.2% received maintenance ICS, 56.3% ICS/FF as needed and 11.6% SABA as needed; 57.4%, 61.2% and 54.9%, respectively, had well-controlled asthma (ACQ-5/ACT definition). After 6 months, changes in mean FEV_1_ were small in the maintenance ICS and ICS/FF as needed groups, whereas there was a decline in FEV_1_ in the SABA as needed group. ACQ-5 total score improved from baseline in all three groups; 0.4%, 0.4% and 2.0% patients, respectively, had a severe exacerbation during the study.

**Conclusions:**

More patients received ICS/FF as needed than SABA as needed, suggesting that physicians are aware of the latest treatment recommendations. This real-world study provides additional support to the use of ICS/FF as needed as preferred treatment for patients with mild asthma, whereas SABA as needed was associated with a fall in lung function and more severe exacerbations.

## Shareable abstract

**More patients received ICS/FF as needed than SABA in PRIME, suggesting that physicians are aware of the latest treatment recommendations. The results provide additional support to ICS/FF as needed as a treatment option for patients with mild asthma.** https://bit.ly/3xCYltL

## Introduction

There have been many changes to mild asthma therapy recommendations over the last decade. Up until 2018, for adults and adolescents with Step 1 asthma the Global Initiative for Asthma (GINA) recommended a short-acting β_2_-agonist (SABA) taken as needed; maintenance low-dose inhaled corticosteroid (ICS) plus SABA as needed was recommended for Step 2 [1]. In their 2019 update, GINA no longer recommended SABA alone at Step 1, but instead advocated low-dose ICS/formoterol fumarate (ICS/FF) as needed [2]. The preferred Step 2 controller became either low-dose ICS/FF as needed or low-dose maintenance ICS plus ICS/FF as needed as reliever (with SABA as needed an alternative reliever). Although ICS/FF as needed as sole anti-inflammatory reliever (AIR-only) has been approved for adolescents and adults with mild asthma in >50 countries worldwide, it is important to note that use of ICS/FF as AIR-only is still “off-label” in the 27 countries of the European Union. Despite this, AIR-only instead of SABA as needed or ICS maintenance treatment in patients with mild asthma is also consistent with recent European Respiratory Society (ERS) guidelines [3].

GINA recommendations continued to evolve, and since 2021 the preferred therapy (“Track 1”) no longer differentiates between Step 1 and 2, with low-dose ICS/FF as needed the recommended controller and reliever option for both steps [4]. However, the alternative (“Track 2”) recommendations were an ICS taken whenever a SABA is used at Step 1, with low-dose maintenance ICS plus SABA as needed at Step 2 [4]. In 2023, the Track 2 Step 2 reliever recommendations changed to either SABA or ICS/SABA as needed (both in addition to low-dose maintenance ICS) [5].

One of the reasons for the change from SABA as needed to AIR-only was the NovelSTART study, in which patients with mild asthma using ICS/FF as needed had a lower annualised exacerbation rate than those using SABA as needed, and a similar rate to those using maintenance ICS plus SABA as needed [6]. Furthermore, those using ICS/FF as needed had a lower number of severe exacerbations than the other two groups. Other data supporting AIR-only rather than maintenance ICS in mild asthma come from SYGMA-1, SYGMA-2 and PRACTICAL, in which ICS/FF as needed in mild asthma (or mild-to-moderate asthma in the case of PRACTICAL) was similarly effective to maintenance ICS in terms of severe exacerbations, while requiring a lower ICS dose [7–9].

Given the evolution of treatment recommendations, it is important to collect data on how patients with so-called mild asthma are being treated in the real world. In addition, it is valuable to collect data on patient outcomes, including symptoms, lung function, asthma control and exacerbations to evaluate disease impact. Importantly, mild asthma does not mean absence of risk; indeed, patients with mild asthma can experience exacerbations and can have poor quality of life [10]. The observational PRIME study sought to address these key questions, by recruiting patients who met the definition of mild asthma (*i.e.*, receiving GINA Step 1 or 2 medication) and who were receiving standard clinical care.

## Materials and methods

This was an international, observational, prospective cohort study. All treatments were prescribed in accordance with local clinical practice (including off-label use), and the only data collected were those derived from standard care. Virtual or remote visits were acceptable (except for the enrolment visit) if this represented standard practice at the site.

Sites were selected on the basis of a feasibility assessment that: investigated the frequency of standard clinic visits; determined the planned assessments formed part of routine clinical practice; and ensured they treated patients with mild asthma. To minimise bias, sites were asked to recruit all eligible patients, which was adults with a confirmed asthma diagnosis (including newly diagnosed) receiving GINA Step 1 or 2 treatment (*i.e.*, mild asthma), specifically: as-needed SABA alone; as-needed ICS/FF alone; or low-dose maintenance ICS plus as-needed SABA or ICS/FF, but without any maintenance long-acting bronchodilator. Minimal exclusion criteria were applied: enrolment in an interventional clinical trial; use of experimental treatments in the previous 3 months; pregnant or breast feeding; unable to complete questionnaires or an eDiary; an asthma exacerbation in the 4 weeks prior to entry; or a diagnosis of COPD. All patients provided written informed consent prior to any study-related procedure.

The follow-up period was 6 months (with a window for the final visit of 5–9 months), during which any asthma treatment was permitted. At enrolment, demographics and the occurrence of asthma exacerbations in the previous 12 months was recorded. In addition, if available as part of routine practice, results were collected from spirometry tests and patient-reported outcomes (Asthma Control Test (ACT), Asthma Control Questionnaire 5 item (ACQ-5) and/or mini Asthma Quality of Life Questionnaire (mini-AQLQ)). After 6 months, available spirometry and patient-reported outcomes data were collected, together with the occurrence of asthma exacerbations and treatment-related adverse events. Severe exacerbations were defined as worsening of asthma leading to: use of systemic corticosteroids for ≥3 days; emergency department or urgent care visit due to asthma that required systemic corticosteroids; or inpatient admission due to asthma (adapted from Reddel *et al*. [11]). Mild/moderate exacerbations were events reported by the investigator as exacerbations that did not meet the definition of severe. Patients recorded medication use and adherence weekly in an eDiary.

The study was approved by the independent ethics committees at each institution, and was performed in accordance with the Declaration of Helsinki, Good Pharmacoepidemiology Practice and Good Clinical Practice. The study was registered at ClinicalTrials.gov (NCT04598555).

### Outcomes

The primary objective was to describe real-world maintenance and as-needed treatment patterns in adults with mild asthma over the 6-month observational period. To do this, patients were divided into three groups based on medication use at entry: 1) low-dose maintenance ICS, used either alone or in combination with ICS/FF as needed or SABA as needed (“maintenance ICS”; note that ICS/FF was not permitted on entry as maintenance therapy, as this would be GINA Step 3); 2) low-dose ICS/FF as needed without maintenance ICS (“ICS/FF as needed”, *i.e.*, AIR-only); and 3) SABA as needed without maintenance ICS (“SABA as needed”).

Secondary end-points included:

- Clinical characteristics at enrolment
- FEV_1_ over the 6-month observational period
- Asthma control (ACQ-5) over the 6-month observational period
- Asthma exacerbations over the 6-month observational period
- Treatment-related adverse events over the 6-month observational period

### Sample size and statistical methods

Given the primary objective was to quantify the proportion of patients in each of the three treatment groups (maintenance ICS, ICS/FF as needed, and SABA as needed), it was calculated that 980 patients (reduced from an original 1200 patients due to delays in enrolment and impact of the coronavirus disease 2019 (COVID-19) pandemic) would ensure that the width of the 95% confidence interval (CI) of the estimated proportions of patients in each treatment group would be no greater than 7% (*i.e.*, from −3.5% to +3.5%). The width of the CI was the chosen measure of the precision of the estimate.

All analyses are descriptive. Patients with missing data for one or more variables were not excluded from the analyses and their data were not imputed.

## Results

### Participants

**FIGURE 1:**
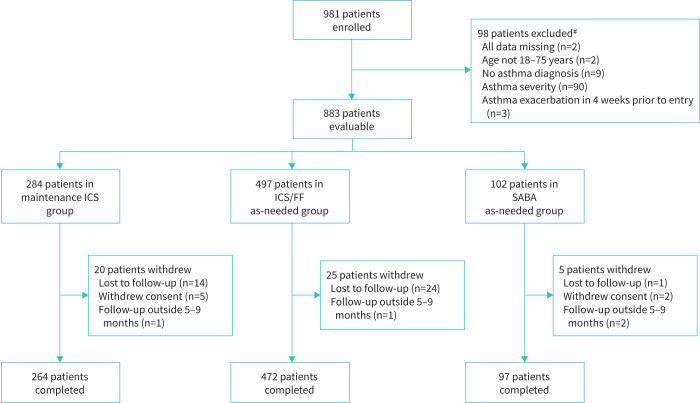
Patient flow through the PRIME study. ICS/FF: inhaled corticosteroid plus formoterol fumarate; SABA: short-acting β_2_-agonist. ^#^: patients could be excluded for more than one reason.

The study was conducted between 25 February 2021 and 2 September 2022 at 56 specialist pulmonologist or allergologist centres (all the patients’ own physicians, providing routine day-to-day care of their asthma) in Germany, Italy, Poland and Spain. Of 981 patients enrolled, 98 were excluded, with the remaining 883 included in these analyses (figure 1, with individual site and country details given in supplementary table S1). Characteristics of the 98 excluded patients were broadly consistent with those included (supplementary table S2). The majority of evaluable patients (833 (94.3%)) completed the 6-month follow-up period, with mean follow-up duration of 6.3 months (range 0–10.5 months).

At enrolment, the majority of patients were receiving ICS/FF as needed only (56.3% of the recruited patients, 95% CI 52.3–60.2%), followed by low-dose maintenance ICS (32.2%, 95% CI 28.5–36.0%) and SABA as needed (11.6%, 95% CI 9.2–14.4%). Most patients in all three groups were receiving the same asthma treatment on entry as during the previous 12 months (63.4–79.4%; table 1) – patients in the ICS/FF as needed group were most likely to have changed to that medication over that period. Approximately 11% were newly treated on enrolment. No patients were using ICS/FF on a “maintenance and reliever therapy” regimen on entry.

**TABLE 1:** Patient baseline characteristics

	Maintenance ICS group	ICS/FF as needed group	SABA as needed group
**Patients**	284	497	102
**Age, years**	44.7±15.4	38.9±14.2	40.3±15.9
**Sex, female**	188 (66.2)	297 (59.8)	69 (67.6)
**Race, Caucasian**	280 (99.6) (n=281)	494 (99.4)	101 (99.0)
**Smoking pack-years**	10.0±12.3	9.2±10.0	8.7±6.3
**Smoking status**			
Nonsmoker	210 (73.9)	362 (72.8)	66 (64.7)
Ex-smoker	53 (18.7)	77 (15.5)	15 (14.7)
Current smoker	21 (7.4)	57 (11.5)	21 (20.6)
**Time since asthma diagnosis years**	9.8±9.9	9.9±10.5	11.5±11.6
**Inhaled asthma treatment in the 12 months prior to enrolment**			
Same as at enrolment	198 (69.7)	315 (63.4)	81 (79.4)
At least one different to therapy at enrolment	56 (19.7)	129 (26.0)	8 (7.8)
None	30 (10.6)	53 (10.7)	13 (12.7)
**Patients who had any skin prick test performed in the 3 years prior to enrolment**	103 (36.3)	200 (40.2)	34 (33.3)
Of those, patients testing positive to any allergen	81 (78.6) (n=103)	173 (86.5) (n=200)	27 (79.4) (n=34)
**FEV_1_ L**	3.04±0.75 (n=277)	3.23±0.87 (n=462)	3.14±0.96 (n=91)
**FEV_1_ % predicted**	92±13 (n=273)	92±15 (n=456)	93±13 (n=91)
**FEV_1_/FVC %**	79±8 (n=277)	77±9 (n=462)	80±8 (n=91)
**ACT total score**	21.4±3.6 (n=283)	21.8±3.0 (n=496)	20.8±3.4
**ACQ-5 score**	0.83±0.91 (n=283)	0.73±0.81 (n=496)	0.87±0.81
**Mini-AQLQ**	5.7±1.0 (n=282)	5.9±0.9 (n=496)	5.5±1.0 (n=101)

Data are presented as mean±sd, n (%) or n. ICS/FF: inhaled corticosteroid plus formoterol fumarate; SABA: short-acting β_2_-agonist; FEV_1_: forced expiratory volume in 1 s; FVC: forced vital capacity; ACT: Asthma Control Test; ACQ-5: Asthma Control Questionnaire, 5 item; mini-AQLQ: mini Asthma Quality of Life Questionnaire.

Patients included in these analyses were predominantly female, Caucasian and nonsmokers, with a mean time since asthma diagnosis of ∼10 years (table 1). Patients in the maintenance ICS group were less likely than the other two groups to be current smokers or to have comorbid chronic rhinitis, and were slightly older (table 1 and supplementary table S3). Just over a third had a skin prick test in the prior 3 years (table 1); most of these tested positive to at least one allergen, most commonly house dust mite (supplementary table S4). When the maintenance ICS group was subgrouped by the type of reliever medication (SABA as needed, ICS/FF as needed, or none), the characteristics were broadly consistent across subgroups, although those receiving ICS plus SABA as needed were on average slightly older, and were more likely to be nonsmokers (supplementary table S5).

At baseline, using the ACQ-5 or ACT definitions, 54.9–61.2% of patients had well-controlled asthma (*i.e.*, ACQ-5 ≤0.75 or ACT >19; table 2). However, the GINA evaluation of asthma control takes into account not only current asthma symptoms (*i.e.*, ACQ-5 or ACT), but also future risk (of exacerbations and lung function impairment). Therefore, the impact of exacerbation history and impaired lung function on asthma control was tested by applying these broader definitions in a stepwise manner, resulting in the proportions falling to 45.1–49.5%.

### Outcomes

Most patients (88.2–92.6%) remained within the same asthma treatment category throughout follow-up (table 3 and supplementary table S6). In the ICS maintenance group (the only group receiving regular medication), 63.0% of patients had adherence >80%.

**TABLE 2:** Application of different definitions of asthma control to evaluate the prevalence of well-controlled asthma in the three different groups at baseline

	Maintenance ICS group	ICS/FF as-needed group	SABA as-needed group
**Patients, n**	284	497	102
**ACQ-5 ≤0.75 or ACT >19**	163 (57.4)	304 (61.2)	56 (54.9)
**ACQ-5 ≤0.75 or ACT >19 and no exacerbations in the 12 months prior to entry**	160 (56.3)	284 (57.1)	52 (51.0)
**ACQ-5 ≤0.75 or ACT >19 and no exacerbations in the 12 months prior to entry and FEV_1_/FVC ≥LLN**	131 (46.1)	246 (49.5)	46 (45.1)

Data are presented as n (%), unless otherwise stated. ICS/FF: inhaled corticosteroid plus formoterol fumarate; SABA: short-acting β_2_-agonist; ACQ-5: Asthma Control Questionnaire, 5 item; ACT: Asthma Control Test; FEV_1_: forced expiratory volume in 1 s; FVC: forced vital capacity; LLN: lower limit of normal (determined for each patient individually).

**TABLE 3:** Shift of asthma treatment category from baseline to study end

Asthma treatment category at study end	Asthma treatment category at enrolment		
	Maintenance ICS group	ICS/FF as needed group	SABA as needed group
**Patients, n**	284	497	102
**Maintenance ICS**	254 (89.4)	2 (0.4)	2 (2.0)
**ICS/FF as needed**	5 (1.8)	460 (92.6)	4 (3.9)
**SABA as needed**	4 (1.4)	1 (0.2)	90 (88.2)
**GINA treatment step 3–5**	19 (6.7)	30 (6.0)	5 (4.9)
**None**	2 (0.7)	0	0
**Other**	0	4 (0.8)	1 (1.0)

Data are presented as n (% within treatment category at enrolment), unless otherwise stated. ICS/FF: inhaled corticosteroid plus formoterol fumarate; SABA: short-acting β_2_-agonist; GINA: Global Initiative for Asthma.

When assessed in patients who did not change asthma therapy throughout the 6-month follow-up, the ICS/FF as needed group used a mean of 0.5 puffs·day^−1^ medication (median (25th to 75th percentile) 0.2 (0.0–0.6)), compared with 0.4 puffs·day^−1^ (0.1 (0.0–0.5)) in the SABA as needed group. As needed medication was used on average <2 days·week^−1^ by 70.3% patients in the ICS/FF as needed group, and by 86.2% in the SABA as needed group. The mean cumulative ICS dose (as beclometasone dipropionate equivalent) was 25 620 µg in the maintenance ICS group, compared with 6634 µg in the ICS/FF as needed group.

**FIGURE 2:**
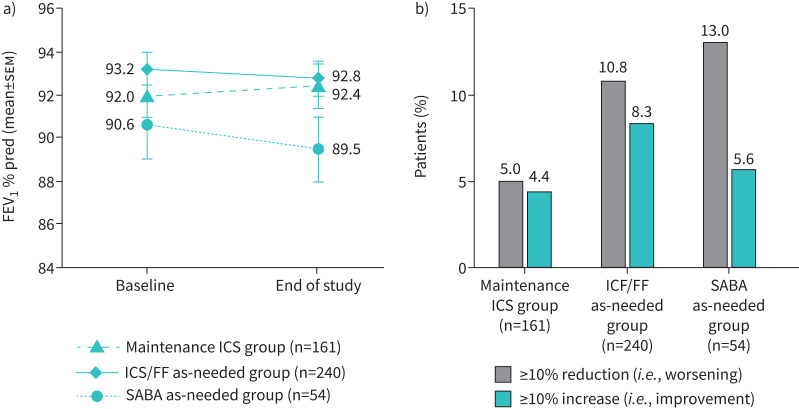
Lung function (forced expiratory volume in 1 s (FEV_1_)) changes over the duration of the study. Data were analysed in patients who did not change therapy over the follow-up period. Data are presented as a) mean±sem and b) categorical analysis, specifically the proportion of patients in each treatment group with a ≥10% reduction (*i.e.*, worsening) or a ≥10% increase (*i.e.*, improvement) from baseline in FEV_1_ at 6 months. % pred: % predicted; ICS/FF: inhaled corticosteroid plus formoterol fumarate; SABA: short-acting β_2_-agonist.

**FIGURE 3:**
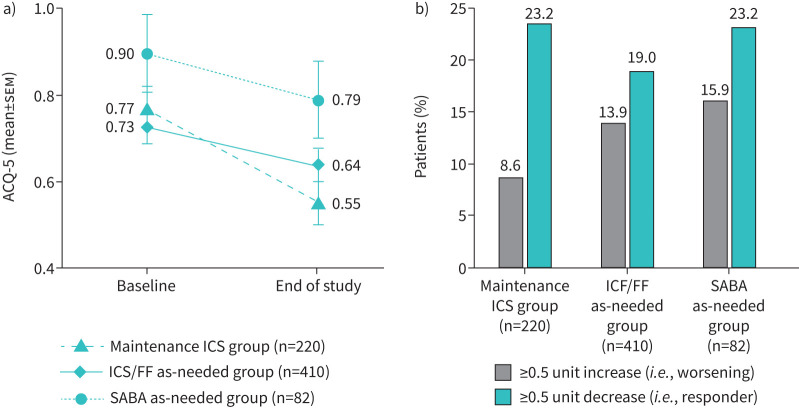
Asthma control (Asthma Control Questionnaire, 5 item (ACQ-5)) changes over the duration of the study, presented as means and the proportion of “responders”. Data were analysed in patients who did not change therapy over the follow-up period. Data are presented as a) mean±sem and b) categorical analysis, specifically the proportion of patients in each treatment group with a ≥0.5 unit increase (*i.e.*, worsening) or a ≥0.5 unit decrease (*i.e.*, improvement) from baseline in ACQ-5 at 6 months. ICS/FF: inhaled corticosteroid plus formoterol fumarate; SABA: short-acting β_2_-agonist.

**FIGURE 4:**
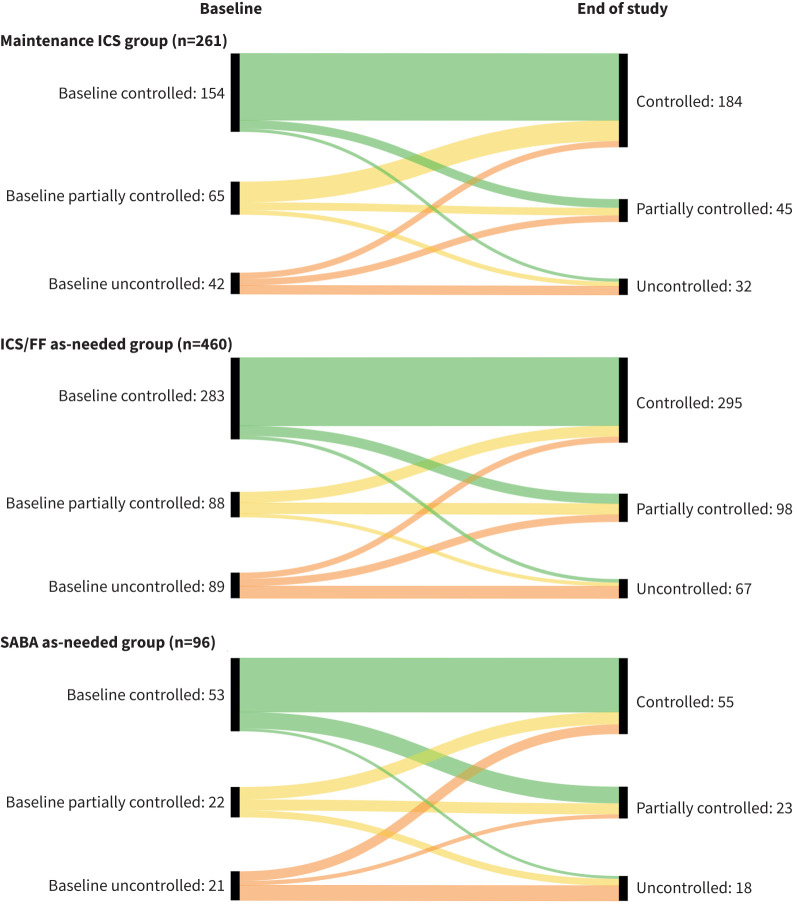
By-patient changes in asthma control category, defined according to Asthma Control Questionnaire (5 item) (controlled: <0.75; partially controlled: ≥0.75 and ≤1.5; uncontrolled: >1.5). Data were analysed in all patients. ICS/FF: inhaled corticosteroid plus formoterol fumarate; SABA: short-acting β_2_-agonist.

In patients not changing therapy over follow-up, changes from baseline in FEV_1_ at study end were small in the maintenance ICS and ICS/FF as needed groups, with similar proportions of patients in the two groups having improvements and worsening (when analysed in categories of ≥10% increase and ≥10% reduction from baseline, respectively; figure 2). In the SABA as needed group, there was a mean FEV_1_ decline, with more than twice as many patients having a worsening than an improvement. The baseline ACQ-5 total score was lowest (*i.e.*, best) in the ICS/FF as needed group; scores in all three treatment groups improved (*i.e.*, decreased) over the follow-up period, with more patients having an ACQ-5 response than worsening in all groups (figure 3). When analysed by ACQ-5 control category, most patients in all three treatment groups did not change category over the follow-up period (figure 4).

**FIGURE 5:**
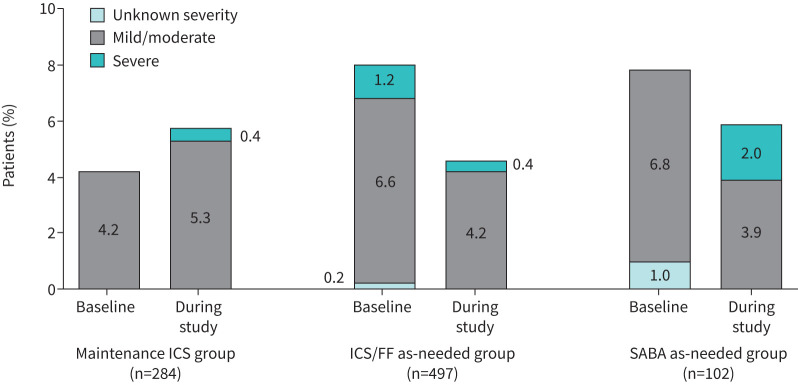
Occurrence of asthma exacerbations during the 12-month period prior to baseline and during the 6-month follow-up period. Data are missing on exacerbations in the previous 12 months for one patient in the maintenance inhaled corticosteroid (ICS) group and two in the ICS/formoterol fumarate (FF) as-needed group; for this figure, it is assumed that these patients did not have an exacerbation over this period. SABA: short-acting β_2_-agonist.

Most patients did not exacerbate either during the 12 months prior to baseline or over the 6-month study period (figure 5). The highest proportion of patients to experience a severe exacerbation during the study was in the SABA as needed group (2.0%).

### Safety

Other than exacerbations, the only non-serious adverse events recorded were those considered related to asthma treatment. The only such event was oral candidiasis, in a patient in the maintenance ICS group. No patients died during the study, and the only serious adverse event reported was an asthma exacerbation, in a patient in the ICS/FF as needed group, that was not considered related to treatment, with the patient completing the study.

## Discussion

This is the first study to provide real-world data on the use of low-dose ICS/FF as needed in patients with mild asthma, helping to address the research needs listed in an American Thoracic Society official statement [12]. Prescribed treatment choice appeared to be broadly consistent with current GINA and ERS guidelines, with more patients using ICS/FF as needed only than SABA as needed only. While symptoms appeared stable across all groups, lung function and asthma control were stable only in those on regular ICS or using ICS/FF as needed (AIR-only), with few patients exacerbating, either in the prior 12 months or over the 6-month observational period. In contrast, in the SABA as needed group, there was a decline in FEV_1_, and 2.0% of patients experienced a severe exacerbation during the follow-up period. There was only one treatment-related adverse event (in the maintenance ICS group), although this study was not designed to evaluate the safety or tolerability of the treatments administered.

The biggest beneficial effect of ICS in patients with asthma is on preventing exacerbations. In NovelSTART there was a 51% lower rate of exacerbations in the ICS/FF as needed group than in the SABA as needed group [6], and in SYGMA-1, there was a 64% reduction in the rate of severe exacerbations in the ICS/FF as needed group compared with the SABA as needed group [7]. The PRIME population demonstrated a lower likelihood of exacerbations than either the NovelSTART or SYGMA-1 populations, potentially as a consequence of COVID-19 social distancing and “lockdown” measures reducing the incidence of virus-induced acute asthma during the period of the study [13, 14]. In addition, it is possible that the PRIME population had less severe asthma than the other studies – for example, whereas six patients (0.7% overall) in PRIME had a severe exacerbation in the year prior to entry (all in the ICS/FF as needed group), this was the case for 7.3% and 19.7% of patients in NovelSTART and SYGMA-1, respectively [6, 7]. As a consequence, it would be very hard to show a positive impact on exacerbations, especially given the shorter (*i.e.*, 6-month) follow-up of PRIME. Despite this, the highest proportion of patients with a severe exacerbation during the PRIME follow-up period was in the SABA as needed group. Importantly, the similar occurrence of exacerbations in the maintenance ICS and the ICS/FF as-needed groups in PRIME is consistent with the results of NovelSTART, SYGMA-1 and SYGMA-2 [6–8], which, together with the decline in lung function in the SABA as needed group, also provide support to GINA and ERS recommendations on the preferential use of ICS/FF as needed in adults with mild asthma [3, 5]. The association between exacerbation frequency and the loss in lung function is well described even in those with “mild” asthma [15].

A notably large proportion of patients entering this study had a significant disease burden (in terms of ACQ-5/ACT), despite being managed as having mild asthma. Indeed, when ACQ-5/ACT was combined with exacerbation history and lung function, fewer than 50% of patients had well-controlled asthma on entry to the study. This therefore suggests that these patients are being undertreated, such that they are being perceived as having milder asthma than is actually the case – and potentially also that the patient-reported outcome measurements ACQ-5 or ACT (which evaluate only symptom control in the prior week or month, respectively) do not appropriately assess the level of control and future risk in this population. Despite this poor overall level of asthma control on entry, 88.2–92.6% of patients did not change treatment during the study. Given the observational nature of the study and the duration of the observational period, the reasons for not changing therapy are unclear (the protocol did not limit treatment changes), and it is possible that medication may have been adjusted at the end-of-study visit. In addition, adherence to maintenance therapy with low-dose ICS was high in comparison to other observational studies that collected data electronically (63.0% of patients in PRIME had adherence >80% *versus* 24.6–34.3% in a prior study [16, 17]). However, it is important to note that this was self-declared adherence (from the eDiary), with patients receiving a weekly reminder to record their medication use.

Use of SABA as needed and ICS/FF as needed was low, averaging one puff every 2 days, with more than three-quarters of the overall population using reliever medication <2 days·week^−1^. As a consequence of this low use, the cumulative ICS dose was markedly lower in the ICS/FF as needed group than in the maintenance ICS group (6634 *versus* 25 620 µg).

Given the fact that use of ICS/FF on an as-needed only basis (AIR-only) is off-label in the four countries involved in the study, limited conclusions can be drawn on overall compliance with guidelines-based treatment recommendations, although the relatively small proportion of patients receiving SABA alone suggests that at least these investigators are aware of the risks of not including an anti-inflammatory therapy. Other limitations of the study include a potential selection bias, since the sites were not randomly selected to represent asthma care in a specific country, and the sites that were involved had an interest in the management of asthma, given all investigators were specialist allergologists or pulmonologists, not primary care physicians (although they were providing routine day-to-day care of the patients’ asthma). However, inclusion of sites from four countries improves the generalisability of the findings, as does the request to sites to recruit all eligible patients. In addition, although sites were asked to recruit patients in a consecutive manner, we do not have any evidence that this was actually the case. Furthermore, given this was an observational study, the only data available were those from standard care, and there was limited opportunity to follow-up on missing data, or to query potentially incorrect data. To minimise the risk of missing data, sites were selected that routinely have 6-monthly follow-up visits, and that use the various questionnaires as part of their standard of asthma care.

### Conclusion

More patients received ICS/FF as needed than SABA as needed in this study, suggesting that physicians are aware of the latest GINA recommendations for the care of patients with mild asthma. However, the high proportion of patients with uncontrolled, yet so-called “mild” asthma (and the lack of changes in treatment category over the follow-up period) highlights the need for further improvements in their care and potentially for more sensitive tools to help detect uncontrolled asthma in this population.

Although the study was not designed to compare treatment groups, with patients not randomised to treatment, this real-world study provides additional support to the use of ICS/FF as needed as a treatment option for patients with mild asthma, with these patients overall tending to gain more benefit from treatment than those receiving SABA as needed, consistent with the results of prior randomised clinical trials.

## Supplementary material

10.1183/23120541.00174-2024.Supp1 **Please note:** supplementary material is not edited by the Editorial Office, and is uploaded as it has been supplied by the author.
Supplementary material 00174-2024.SUPPLEMENT

## Data Availability

Chiesi commits to sharing with qualified scientific and medical researchers, conducting legitimate research, the anonymised patient-level and study-level data, the clinical protocol and the full clinical study report of Chiesi Farmaceutici SpA-sponsored interventional clinical trials in patients for medicines and indications approved by the European Medicines Agency and/or the US Food and Drug Administration after 1 January 2015, following the approval of any received research proposal and the signature of a Data Sharing Agreement. Chiesi provides access to clinical trial information consistently with the principle of safeguarding commercially confidential information and patient privacy. Other information on Chiesi's data sharing commitment, access and research request's approval process are available in the Clinical Trial Transparency section of www.chiesi.com/en/research-and-development/
