# Supplementary material for: Real-world use of inhaled corticosteroid/formoterol as needed in adults with mild asthma: the PRIME study
Source: ERJ Open Res. 2024 Sep 30;10(5):00174-2024. doi: 10.1183/23120541.00174-2024 (PMC11440373; doi:10.1183/23120541.00174-2024)
Supplement: Supplementary file 1 [file 00174-2024.SUPPLEMENT.pdf]

Real-world use of inhaled corticosteroid/formoterol as-needed in adults with mild asthma: The PRIME study

Guy Brusselle, Francesco Blasi, Christian Gessner, Piotr Kuna, Peter Wark, Glauco Cappellini, Emilie Oosterom, Marielle Van Der Deijl, Enrica Bucchioni, Eva Topole

Supplementary results

Supplementary Table S1. Site details, including number of patients evaluable and enrolled.

Site number	Site details	Evaluable patients (n=883)		Enrolled patients (n=981)	
		**n**	**%**	**n**	**%**
**Germany**					
DE-001	Salvus – Klinische Studien GmbH – Dr Deckelmann	0	0.0	1	0.1
DE-002	RespiRatio / Lungenpraxis Schleswig – Dr Deimling	37	4.2	40	4.1
DE-003	Praxis Pneumologie und Allergologie Dr Ginko – Dr Ginko	20	2.3	21	2.1
DE-004	Zentrum für ambulante Pneumologische Forschung Marburg GbR – Dr Mronga	36	4.1	36	3.7
DE-005	Praxis für Pneumologie und Innere Medizin – Herr. Xanthopoulos	26	2.9	28	2.9
DE-007	POIS Sachsen GmbH iG– PD. Dr Geßner	1	0.1	1	0.1
DE-008	Lungenarztpraxis Hellersdorf – Herr. Driemert	21	2.4	22	2.2
DE-009	Praxis Dr Jansen – Dr Jansen	58	6.6	59	6.0
DE-010	Studienzentrum Dr Schlenska – Dr Schlenska	3	0.3	4	0.4
DE-012	Lungenärzte am Rundfunkplatz – Dr Avsar	11	1.2	17	1.7
**Germany total**		**213**	**24.1**	**229**	**23.3**
**Spain**					
ES-002	Hospital Universitario La Princesa – Ph MD Cisneros Serrano	4	0.5	4	0.4
ES-003	Hospital Universitario Dr Peset – MD Martínez Moragon	14	1.6	15	1.5
ES-004	Hospital Universitario La Paz – MD PhD Domínguez Ortega	5	0.6	5	0.5
ES-005	Hospital Germans Trias I Pujol – Dr Roger Reig	9	1.0	10	1.0
ES-007	Complejo asistencial Universitario de Salamanca – Dr Moreno Rodilla	5	0.6	6	0.6
ES-008	Fundación Jiménez Díaz – MD PhD Sastre Dominguez	7	0.8	8	0.8
ES-009	Hospital de la Santa Creu I Sant Pau – Dr PhD Plaza Moral	7	0.8	10	1.0

Site number	Site details	Evaluable patients (n=883)		Enrolled patients (n=981)	
		n	%	n	%
ES-011	Hospital Universitario Marqués de Valdecilla – Dr García Rivero	5	0.6	5	0.5
ES-014	Hospital El Pilar – Dr PhD Rubinstein Aguñin	13	1.5	19	1.9
**Spain total**		**69**	**7.8**	**82**	**8.4**
**Italy**					
IT-001	UOC Pneumologia Fondazione Cà Granda Ospedale Maggiore Policlinico – Prof. Blasi	5	0.6	7	0.7
IT-003	UO Pneumologia Riabilitativa ICS Maugeri – Dr Visca	9	1.0	11	1.1
IT-005	UO Allergologia Ospedale S.Maria della Speranza – Prof. Patella	40	4.5	40	4.1
IT-006	SC MAR Univ A.O.U. Ospedali Riuniti Foggia – Dr Scioscia	9	1.0	10	1.0
IT-007	Malattie Apparato Respiratorio A.O.U. Modena Policlinico – Prof. Beghè	4	0.5	4	0.4
IT-008	Pneumologia Policlinico Universitario Mater Domini – Prof. Pelaia	5	0.6	5	0.5
IT-009	U.O. Malattie Apparato Respiratorio Pneumologia Clinica e Interventistica AOU Policlinico stabilim San Pietro – Prof. Fois	9	1.0	9	0.9
IT-010	Medicina del Lavoro, Mal.Respiratorie e Tossicologia Azienda Ospedaliera – Dr Gambelunghe	3	0.3	3	0.3
IT-012	UOC Clinica Pneumologica A.O. dei Colli P.O. Monaldi – Prof. Bianco	10	1.1	10	1.0
IT-013	UO Pneumologia A.O.U. Pisana P.O. Cisanello – Dr Dente	5	0.6	6	0.6
IT-015	UOC Pneumologia Policlinico Univ. A. Gemelli – Dr Bonini	28	3.2	31	3.2
IT-016	SSD Intertiziopatie e Malattie del Polmone A.O.U. San Luigi Gonzaga – Prof. Ricciardolo	30	3.4	34	3.5
IT-017	Pneumologia Riabilitativa ICS Maugeri – Dr Maniscalco	7	0.8	8	0.8
IT-018	USD Allergologia AOUI Policlinico Rossi – Prof. Senna	17	1.9	18	1.8

Site number	Site details	Evaluable patients (n=883)		Enrolled patients (n=981)	
		n	%	n	%
IT-019	UOC Pneumologia Ospedale Bellaria – Dr Rocca	16	1.8	16	1.6
IT-020	UOC Pneumologia A.O.U. Sant'Andrea – Prof. Ricci	13	1.5	14	1.4
IT-021	Ambulatorio Pneumologia PTA Ospedale Busacca – Dr Cannata	4	0.5	21	2.1
IT-022	Pneumologia Univ. A.O.U. Città della Salute e della Scienza – Dr Solidoro	4	0.5	4	0.4
IT-025	Allergologia e Immunologia clinica Policlinico Casula A.O.U. Cagliari – Prof. Del Giacco	13	1.5	19	1.9
IT-026	SOS Allergologia A.O.U. Ospedali Riuniti – Dr Bilò	18	2.0	20	2.0
IT-028	Fisiopatologia Respiratoria Ospedale Ecclesiastico Miulli – Dr Schino	47	5.3	60	6.1
IT-029	Malattie Respiratorie e Trapianto Polmonare A.O.U. Senese Policlinico Le Scotte – Prof. Bargagli	11	1.2	13	1.3
IT-030	UO Pneumologia – Istituti Clinici Scientifici Maugeri – Dr Aliani	7	0.8	8	0.8
**Italy total**		**314**	**35.6**	**371**	**37.8**
**Poland**					
PL-001	Poradnia Alergologii i Chorób Płuc, SPZOZ Uniwersytecki Szpital Kliniczny nr 1 im Norberta Barlickiego – Prof. Kuna	38	4.3	39	4.0
PL-003	Praktyka lekarska Marzena Justyna Mierzejewska – Dr n.med. Mierzejewska	17	1.9	18	1.8
PL-004	CDT Medicus – Dr n.med. Kwaśniewski	4	0.5	5	0.5
PL-005	Grażyna Pulka Centrum Medyczne ALL-MED – Dr n.med. Pulka	10	1.1	10	1.0
PL-006	PPL PS MAGMED – lek.med. Napora-Grabowska	25	2.8	25	2.5
PL-009	Centrum Alergologii Sp. Z o. o – Dr n.med. Krupa-Borek	16	1.8	16	1.6
PL-010	M2M Badania Kliniczne sp. Z o. o – lek.med. Płaczek	38	4.3	38	3.9

Site number	Site details	Evaluable patients (n=883)		Enrolled patients (n=981)	
		**n**	**%**	**n**	**%**
PL-011	Lekarze Specjaliści J.Małolepszy I Partnerzy – Dr n.med. Wytrychowski	15	1.7	17	1.7
PL-013	SNZOZ Poradnia Specjalistyczna MedMed, Ul. Brzechwy 7a – lek.med. Kuczyńska	5	0.6	6	0.6
PL-014	Indywidualna Specjalistyczna Praktyka Lekarska Marzenna Tarnowska-Matusiak – lek.med. Drążewska	20	2.3	21	2.1
PL-015	Prywatny gabinet Pulmonologiczny at premises of Amicmed Medical Center – lek.med. Nowacka-Apiyo	20	2.3	20	2.0
PL-016	Sławomir Garbicz NZOZ Poradnia Chorób Płuc I Alergologii – lek.med. Garbicz	15	1.7	19	1.9
PL-017	Gabinet Pulmonologiczny dr n.med. Beata Janiszewska - Drobińska – Dr n.med. Janiszewska-Drobińska	10	1.1	10	1.0
PL-018	Prywatny Gabinet Lekarski Ewa Gawrońska-Ukleja – Dr n.med. Gawrońska-Ukleja	54	6.1	55	5.6
**Poland total**		**287**	**32.5**	**299**	**30.5**

Supplementary Table S2. Characteristics of patients included and excluded from the analyses.

	**Included in analyses (n=883)**	**Excluded from analyses (n=98)**
Age, years	40.9 (15.0)	43.4 (15.1) (n=94)
Sex, female	554 (62.7%)	55 (58.5%)
Race, Caucasian	875 (99.4%) (n=880)	93 (94.9%)
Smoking status		(n=91)
Non-smoker	638 (72.3%)	63 (69.2%)
Ex-smoker	145 (16.4%)	18 (19.8%)
Current smoker	99 (11.2%)	10 (11.0%)
FEV_1, L	3.16 (0.85) (n=830)	3.02 (0.91) (n=89)
FEV_1, % predicted	92 (14) (n=820)	89 (16) (n=87)
FEV_1/FVC, %	78 (9) (n=830)	78 (10) (n=89)
ACQ-5 score	0.78 (0.84) (n=881)	N/A

Data are mean (standard deviation), or number (percent). ICS/FF, inhaled corticosteroid plus formoterol fumarate; SABA, short-acting β_2-agonist; FEV_1, forced expiratory volume in 1 sec; FVC, forced vital capacity; ACQ-5, Asthma Control Questionnaire, 5 item.

Supplementary Table S3. Additional patient baseline characteristics.

	**Maintenance ICS group (n=284)**	**ICS/FF as-needed group (n=497)**	**SABA as-needed group (n=102)**
Body-mass index category			
Underweight (<18.5 kg/m^2)	8 (2.8%)	11 (2.2%)	6 (5.9%)
Normal weight (18.5–25 kg/m^2)	115 (40.5%)	231 (46.5%)	46 (45.1%)
Overweight (25–<30 kg/m^2)	91 (32.0%)	163 (32.8%)	31 (30.4%)
Obese (≥30 kg/m^2)	69 (24.3%)	84 (16.9%)	19 (18.6%)
Unknown	1 (0.4%)	8 (1.6%)	0
Selected comorbidities			
Chronic rhinitis	85 (29.9%)	243 (48.9%)	37 (36.3%)
Gastro-oesophageal reflux disease	24 (8.5%)	50 (10.1%)	3 (2.9%)
Sinusitis	13 (4.6%)	25 (5.0%)	4 (3.9%)
Atopic dermatitis	9 (3.2%)	28 (5.6%)	2 (2.0%)
Nasal polyps	4 (1.4%)	14 (2.8%)	0

Data are mean (standard deviation), or number (percent). ICS/FF, inhaled corticosteroid plus formoterol fumarate; SABA, short-acting β_2-agonist.

Supplementary Table S4. Patients testing positive to allergens (as a percentage of those who had a skin prick test in the prior 3 years).

	**Maintenance ICS group (n=103)**	**ICS/FF as-needed group (n=200)**	**SABA as-needed group (n=34)**
Any	81 (78.6%)	173 (86.5%)	27 (79.4%)
Animal mix	2 (1.9%)	7 (3.5%)	0
Aspergillus	5 (4.9%)	5 (2.5%)	2 (5.9%)
Cat hair	22 (21.4%)	63 (31.5%)	8 (23.5%)
Dog hair	13 (12.6%)	52 (26.0%)	4 (11.8%)
Dust mite (*D. pteronyssinus*)	46 (44.7%)	105 (52.5%)	17 (50.0%)
Food mix	1 (1.0%)	6 (3.0%)	1 (2.9%)
Grass mix	44 (42.7%)	115 (57.5%)	16 (47.1%)
Mould mix	5 (4.9%)	18 (9.0%)	1 (2.9%)
Trees	44 (42.7%)	107 (53.5%)	12 (35.3%)
Weed mix	14 (13.6%)	33 (16.5%)	3 (8.8%)
Other(s)	18 (17.5%)	71 (35.5%)	7 (20.6%)

ICS/FF, inhaled corticosteroid plus formoterol fumarate; SABA, short-acting β_2-agonist.

Supplementary Table S5. Patient baseline characteristics of the maintenance ICS group split by type of reliever medication, and of patients excluded from the study.

	**Maintenance ICS group**		
	**ICS plus SABA as-needed (n=204)**	**ICS plus ICS/FF as-needed (n=40)**	**ICS alone (n=40)**
Age, years	45.8 (15.0)	41.4 (15.6)	42.6 (17.0)
Sex, female	142 (69.6%)	20 (50.0%)	26 (65.0%)
Race, Caucasian	202 (99.0%)	38 (95.0%)	40 (100%)
Smoking status			
Non-smoker	156 (76.5%)	29 (72.5%)	25 (62.5%)
Ex-smoker	36 (17.6%)	10 (25.0%)	7 (17.5%)
Current smoker	12 (5.9%)	1 (2.5%)	8 (20.0%)
FEV_1, L	2.98 (0.74) (n=201)	3.20 (0.72) (n=39)	3.25 (0.76) (n=37)
FEV_1, % predicted	92 (14) (n=199)	93 (11) (n=37)	96 (11) (n=37)
FEV_1/FVC, %	79 (8) (n=201)	77 (8) (n=39)	80 (9) (n=37)
ACQ-5 score	0.81 (0.88) (n=203)	0.77 (0.95)	1.03 (1.00)

Data are mean (standard deviation), or number (percent). ICS, inhaled corticosteroid; SABA, short-acting β_2-agonist; FF, formoterol fumarate; FEV_1, forced expiratory volume in 1 sec; FVC, forced vital capacity; ACQ-5, Asthma Control Questionnaire, 5 item.

Supplementary Table S6. Shift of asthma treatment category from baseline to study end.

**Asthma treatment category at study end**	**Maintenance ICS group asthma treatment category at enrolment**		
	**ICS plus SABA as-needed (n=204)**	**ICS plus ICS/FF as-needed (n=40)**	**ICS alone (n=40)**
ICS plus SABA as-needed	191 (93.6%)	0	0
ICS plus ICS/FF	0	28 (70.0%)	0
ICS alone	0	0	35 (87.5%)
ICS/FF as-needed	0	4 (10.0%)	1 (2.5%)
SABA as-needed	4 (2.0%)	0	0
GINA treatment step 3–5	9 (4.4%)	8 (20.0%)	2 (5.0%)
None	0	0	2 (5.0%)
Other	0	0	0

Data are number of patients (% within treatment category at enrolment). ICS, inhaled corticosteroid; SABA, short-acting β_2-agonist; FF, formoterol fumarate; GINA, Global Initiative for Asthma.
